# Beyond physical accessibility, bypassing health facilities offering caesarean section: insights from women in Dakar’s slums

**DOI:** 10.1136/bmjopen-2024-088606

**Published:** 2025-03-22

**Authors:** El Hadji Malick Sylla, Ndeye Awa Fall, Winfred Dotse-Gborgbortsi, Arsène Brunelle Sandie, Barrel Sow Gueye, Diarra Bousso Senghor, Birane Cissé, Fadima Yaya Bocoum, Ibrahima Ousmane Sy, Cheikh Faye

**Affiliations:** 1 West Africa Regional Office, African Population and Health Research Center (APHRC), Dakar, Senegal; 2 School of Geography and Environmental Sciences, University of Southampton, Southampton, UK; 3 Geography, Universite Cheikh Anta Diop de Dakar, Dakar, Senegal; 4 Université Cheikh Anta Diop de Dakar, Dakar, Senegal; 5 Institut de Recherche en Sciences de la Sante, Ouagadougou, Centre, Burkina Faso

**Keywords:** Cesarean Section, EPIDEMIOLOGIC STUDIES, Emergency Service, Hospital, Health Services Accessibility

## Abstract

**Abstract:**

**Objective:**

The study examines the geographic accessibility of Comprehensive Emergency Obstetric Care (CEmONC) among women residing in the slums of Dakar.

**Design:**

The study is a cross-sectional geographic analysis of caesarean care utilisation in health facilities offering the service in Dakar.

**Setting:**

The study was conducted in urban slum areas in Dakar.

**Participants:**

763 women living in urban slums who had undergone a caesarean section in six health facilities in Dakar between July and December 2022.

**Outcome measures:**

The proportion of women bypassing the nearest health facility and travel time to health facilities.

**Results:**

Key findings show that most women in Dakar’s urban slums live within 5 min from a health facility offering caesarean services, with an average travel time of 6.3 min. However, 44.3% bypassed nearby facilities, often travelling outside their district. Medical referral was the primary reason for bypassing (43.2%), followed by the search for higher quality care (13.5%) and reliance on family or social networks (14.9%). Only a small proportion (1.4%) cited more affordable treatment costs as a reason for bypassing.

**Conclusion:**

Despite the good geographical accessibility of health facilities offering caesarean sections in Dakar, many women bypass nearby facilities due to medical referrals and the search for higher quality care, resulting in increased travel time and costs. Strengthening the quality and capacity of local health centres in urban slums is crucial to minimising unnecessary bypassing and ensuring timely access to essential obstetric services.

**STRENGTHS AND LIMITATIONS OF THIS STUDY**

- The article highlights the particular challenges faced by women in disadvantaged urban neighbourhoods, as opposed to the more commonly studied rural populations.
- Focus on caesarean sections, whereas previous studies on bypassing of health facilities have mainly addressed delivery without specifying the type.
- We use modelled travel time rather than straight-line distances to determine the accessibility of Comprehensive Emergency Obstetric and Newborn Care (CEmONCs) and the additional travel time taken by bypassing women.
- Our model does not take traffic into account, which could be challenging in urban settings such as Dakar.
- The article focuses only on caesarean section, which limits the generalisability of the results to other forms of obstetric or medical care.

## Introduction

 Physical accessibility to health facilities remains a major challenge in sub-Saharan Africa, where 70% of maternal deaths were recorded in 2020 and where the neonatal mortality rate is the highest in the world.^1^ Distance from health facilities is one of the main factors affecting maternal and neonatal mortality in low-income and middle-income countries.^2^,^4^ It leads to underutilisation of maternal health services leading to increased home deliveries, particularly in the absence of skilled health providers, and a higher risk of maternal deaths.^5 6^ In Kenya, for example, women are willing to give birth in a health facility if it is 2 km away. Beyond this distance, women have the same probability of giving birth at home or in a health facility.^7^ A similar finding has also been documented in Eastern Ghana.^8^ Additionally, findings from Ghana also indicate that women are willing to bypass the closest health facilities and spend more than 2 hours on the road to give birth in other health facilities, in order to benefit from a better quality of service.

Bypassing health facilities is a recurring phenomenon and reflects the inefficiency of a health system. Several studies have addressed this issue and argued that the choice of place of delivery is often explained by the search for better quality care in higher level hospitals when the closest health facilities offer lower quality of care^9 10^ and underskilled staff.^11^ However, most of these studies did not specify the type of delivery (caesarean section vs normal delivery) and focused mainly on rural areas.^12^,^14^ Women in urban slums, despite the perceived advantages of urban life, may experience similar or even greater challenges.^15 16^ In addition, studies have used secondary data from specific research projects such as clinical trials,^17^ routine data,^18^ demographic and health surveys (EDS).^19 20^ The use of primary data, collected through surveys whose main aim is to provide evidence on the problem of accessibility to health facilities and the reasons why women bypass them, is limited.

In order to improve access to emergency obstetric care in Senegal, c-section fees exemption was introduced in 2005 in five pilot regions. The policy was later extended to referral hospitals in other regions, excluding Dakar.^21^,^23^ In 2013, this policy was extended to the Dakar region, where the c-section rate is at 18.4%, compared with 11.4% at the national level.^24^ However, although Dakar has the highest concentration of high-level public health facilities (EPS) and is the wealthiest region in the country,^25^ little conclusive evidence has been produced to understand the access of poor women, mainly located in slums, to these EPS, especially to perform caesarean sections when medically necessary.

This study aims to assess the physical accessibility of women living in the slums of Dakar to emergency caesarean section and to document the factors influencing their choice of health facility. Specifically, we aim to determine the percentage of women who bypass the nearest health facility, understand their reasons and formulate policy/programme recommendations to improve Comprehensive Emergency Obstetric and Newborn Care (CEmONC) services. This pioneering study in Dakar and in West Africa examines expected and observed flows between slums and CEmONC facilities while identifying direct and indirect factors contributing to bypass behaviour.

## Methods

### Study type and area

The study is a spatial, quantitative, cross-sectional and retrospective analysis. Data were collected from health facilities performing caesarean sections and from women who underwent caesarean section between July and December 2022, which is 6 months before the start of the survey. The study area was limited to Dakar and seven of the region’s 10 health districts. The remaining three health districts were excluded due to their very low caesarean section rates. Each surveyed health facility was geo-referenced, with data on indicators enabling comparative and spatial analysis

### Definition of slums

We defined slums as irregular settlements and traditional neighbourhoods, based on the classification provided by Ndiaye^26^ and the Dakar’s 2025 Urban Development Strategy Document.^27^ Initially, we provided the supervisors who processed the delivery rooms and operating theatres registers of the surveyed health facilities with a list of identified slums to help them identify women living in eligible neighbourhoods. We then established the survey quotas for each health facility and coordinated appointments with randomly selected women who agreed to participate in the study (table 1).

**Table 1:** Number of health facilities and slums analysed

District	Frequency (%)
Number of health facilities analysed	
Dakar	12 (66.7)
Guediawaye	2 (11.1)
Pikine	3 (16.7)
Rufisque	1 (5.5)
Number of slums analysed	
Dakar	18 (42.2)
Guediawaye	4 (8.9)
Pikine	15 (33.3)
Rufisque	7 (15.6)

### Data

Two data sources were used in this study. The first was the database from the delivery rooms and operating theatres in six public health facilities, representing 32% of public health facilities performing caesarean sections in Dakar during our study period. The selection criteria included geographical location, type of health facility (hospital or health centre), number of caesarean sections performed and completeness of data on women having given birth by caesarean section between July and December 2022. This period was chosen because it enabled the selection of a representative sample of the target population while avoiding the excessive time required to analyse records spanning a longer period.

A total of 763 women who had undergone a caesarean section in the six target health facilities and who lived in the slums of Dakar were identified. For each woman, we recorded her place of residence and the place where she gave birth by caesarean section. This information was used to estimate the road network distances and travel times covered by women to access emergency caesarean sections.

Among these women, we conducted in-depth surveys with 108 participants, representing 14% of the total. This survey constituted our second data source and aimed to gather information on the sociodemographic characteristics of the women as well as on the reasons that influenced their choice of health facility for their caesarean section.

### Travel time modelling

Motorised travel times were estimated using ArcGIS Online proximity tools from the slum location to the health facilities. For the nearest health facility, we used the ‘Find Nearest’ tool to estimate travel times between slums and the nearest health facility providing caesarean services. We then used the ‘Connect Origins to Destinations’ function to model travel times from slum locations to the health facilities where women underwent caesarean section. Environmental Systems Research Institute (ESRI) estimates the fastest travel times between locations using road network analysis in ArcGIS online with a high-quality cloud-based global road network. In locations like Senegal where ArcGIS lacks real-time traffic data, historical speed data, road conditions and average travel speeds are used to model travel times between locations. The driving times implement rules like one-ways, compliance with local restrictions and other road regulations. The maps were displayed using freely available OpenStreetMap basemap and administrative border outlines from Geoboundaries.^28 29^

### Statistical analyses

Bivariate and multivariate analyses were conducted. The bivariate analysis focused on the number of bypasses per health facility and explanatory reasons. The multivariate analysis used in this study was multiple correspondence factorial analysis (MCA), a tool for examining relationships between two or more qualitative variables without assigning them the roles of dependent and independent variables, as in regression analysis. The variables used to produce the MCA included the health facility where the caesarean section was performed, the woman’s travel time, the administrative location, the name of the slums, the associated health facility and the reasons behind the women’s choice.

## Results

### Geographic accessibility

**Figure 1:**
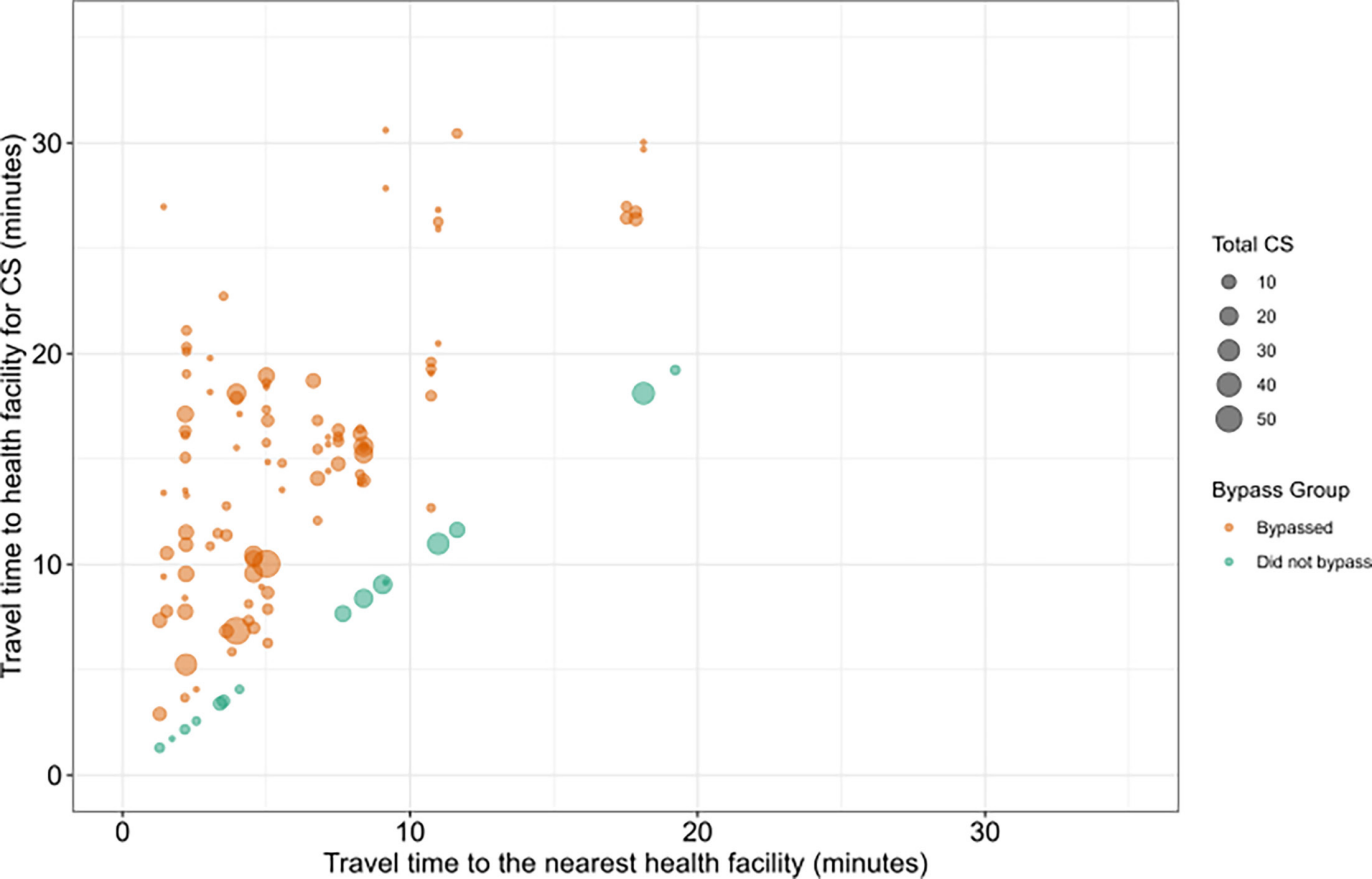
Distance from the slums to the nearest health facility providing caesarean section (n=763). CS, caesarean section.

**Figure 2:**
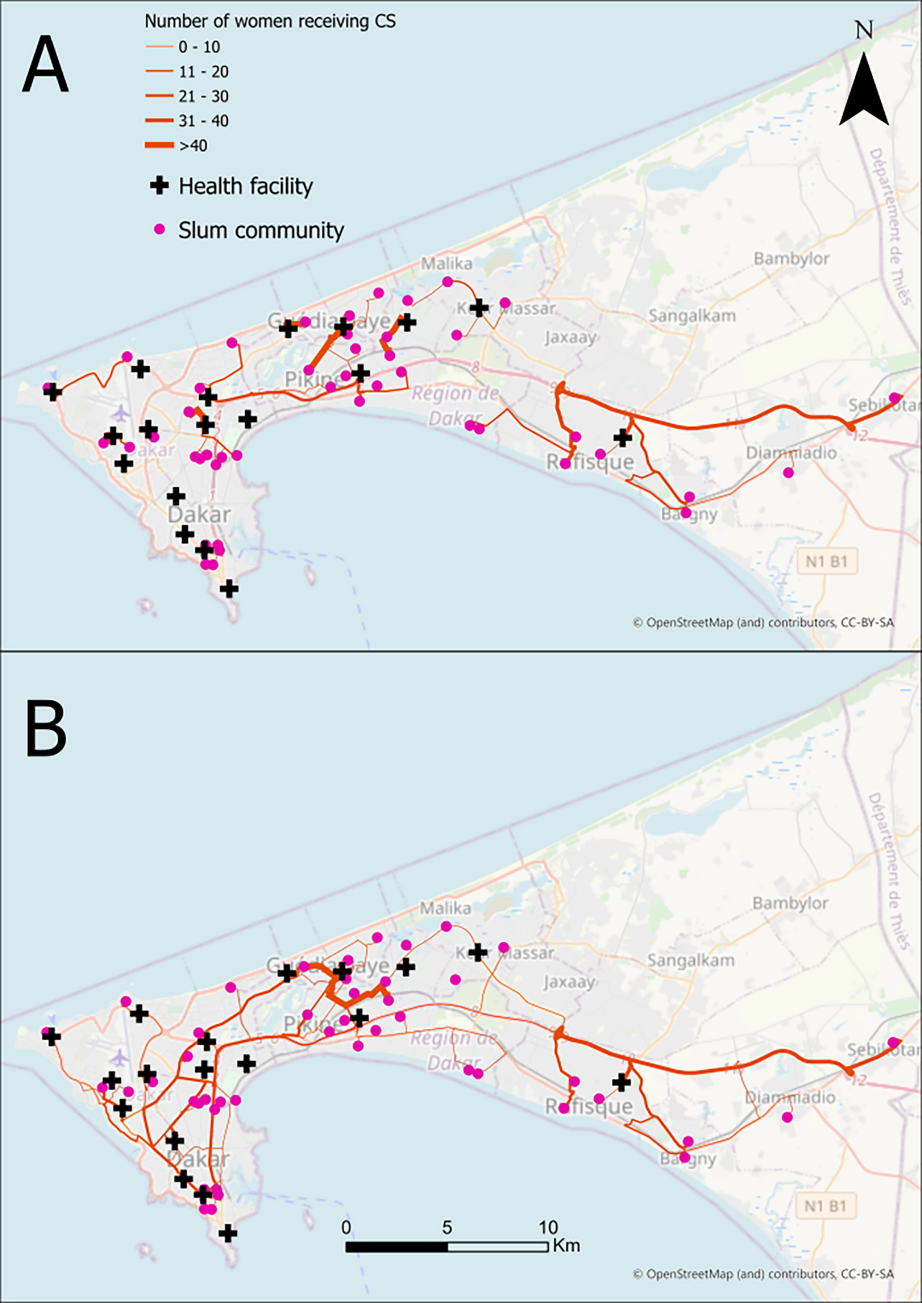
Expected flows (A) versus observed flows (B) in six health facilities surveyed (n=763). CS, caesarean section.

Figure 1 and figure 2A show the expected flows from slum locations to the nearest health facility offering caesarean section services. The average travel time to the nearest health facility providing caesarean services was 6.3 min (SD: 4.6 min), with travel times ranging from 1.3 to 19.2 min. The majority of slums, 27 (60%), were within a 5 min by car of the nearest health facility offering caesarean services, while a few, 4 (8.9%), were more than 15 min away.

Most women, 657 (88.4%), used a health facility within their district, while the remaining women travelled to another district for care. All the women closest to a health facility outside their district were from Pikine. Women travelled from Pikine to Rufisque (10), from Pikine to Dakar (6) and from Pikine to Guédiawaye (70). All the districts assessed had health facilities offering caesarean services.

The average travel time for women whose nearest health facility was within their district was 6 min, compared with 11 min for those who had to travel outside their district to reach the nearest health facility.

Figure 2B shows the actual movement of women from their slum to the health facilities where they underwent caesarean care. The average distance along the road network from the slums to the nearest health facility providing caesarean services was 3.8 km (SD: 4.4 km). The distance ranged from 0.5 to 24.4 km, with a median of 2.3 km (IQR: 2.4). Most slums, 31 (68.9%), were within 2 km of a health facility, compared with only 2 (4%) that were 10 km or more away.

Regarding caesarean service utilisation patterns, 44.3% of women did not use a health facility within their district. Among women who bypassed their nearest health facility, more than half, 309 (51.1%), travelled outside their district for care. In contrast, 138 (87.3%) of those who did not bypass sought caesarean care within their district. Bypassing was more widespread in Dakar than in other districts. In Dakar, 222 (96%) of the women originating from Dakar bypassed a caesarean service within the city. There was also significant movement of women from other districts to Dakar, with 34 women from Guédiawaye, 174 from Pikine and 10 from Rufisque travelling there for caesarean services. Women from Rufisque primarily used a health facility within their district.

The average travel time for women who travelled within their district to seek caesarean services was 10 min, compared with 16 min for those who had to travel outside their district to reach their health facility for care.

### Reason for bypassing caesarean care

**Figure 3:**
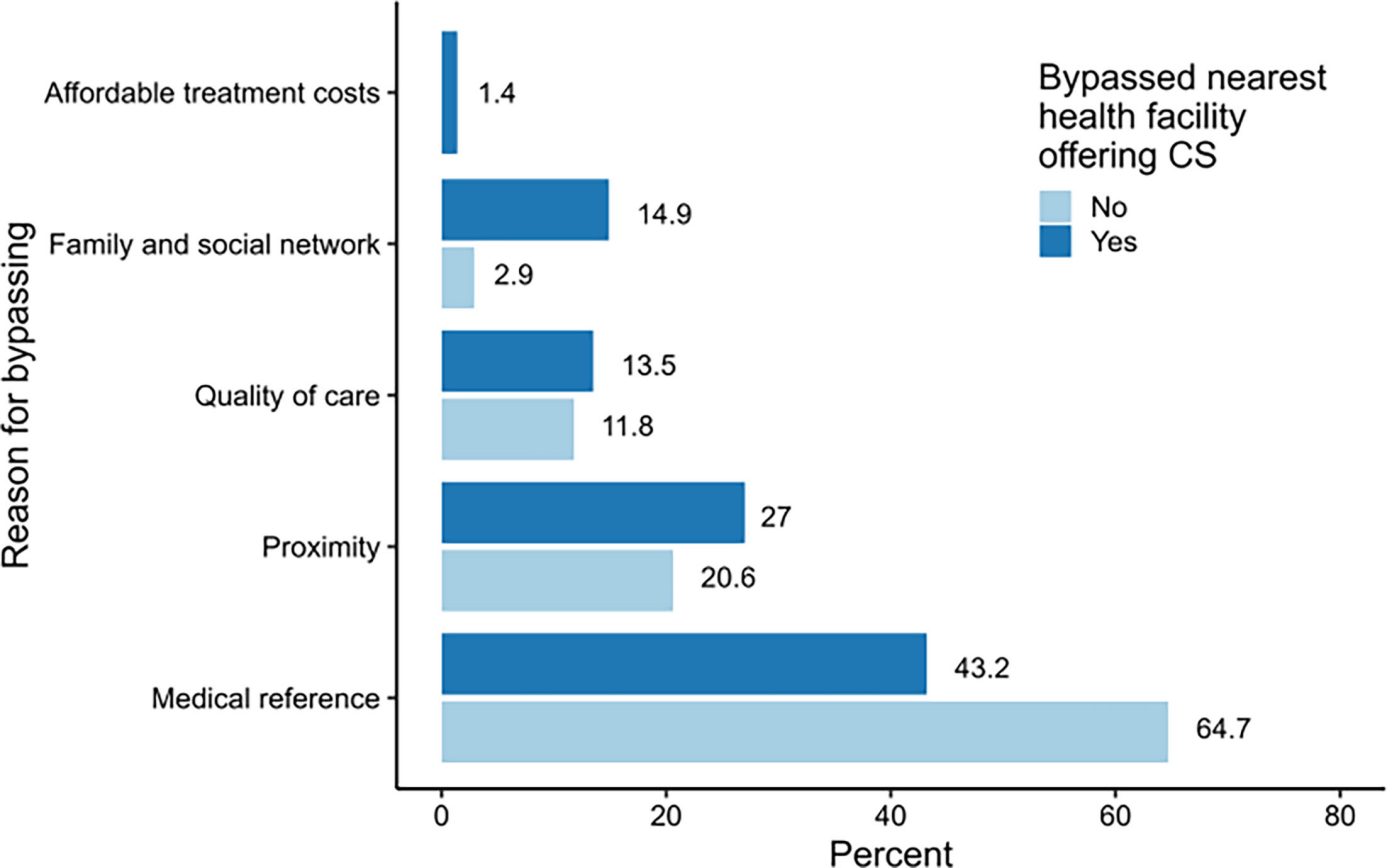
Reason for bypassing (n=108). CS, caesarean section.

Medical referral is the main factor explaining where the caesarean section was performed (figure 3). It represents 64.7% of cases among women who did not bypass the nearest health facility and 43.2% among those who did. Women who bypassed their nearest health facility are more likely to seek higher quality care (13.5%) than those who did not (11.8%). They also rely more on their social or family networks to choose a caesarean section facility (14.9%) than those who did not bypass. A few women who bypassed their nearest health facility cited seeking more affordable treatment costs as their choice (1.4%).

**Figure 4:**
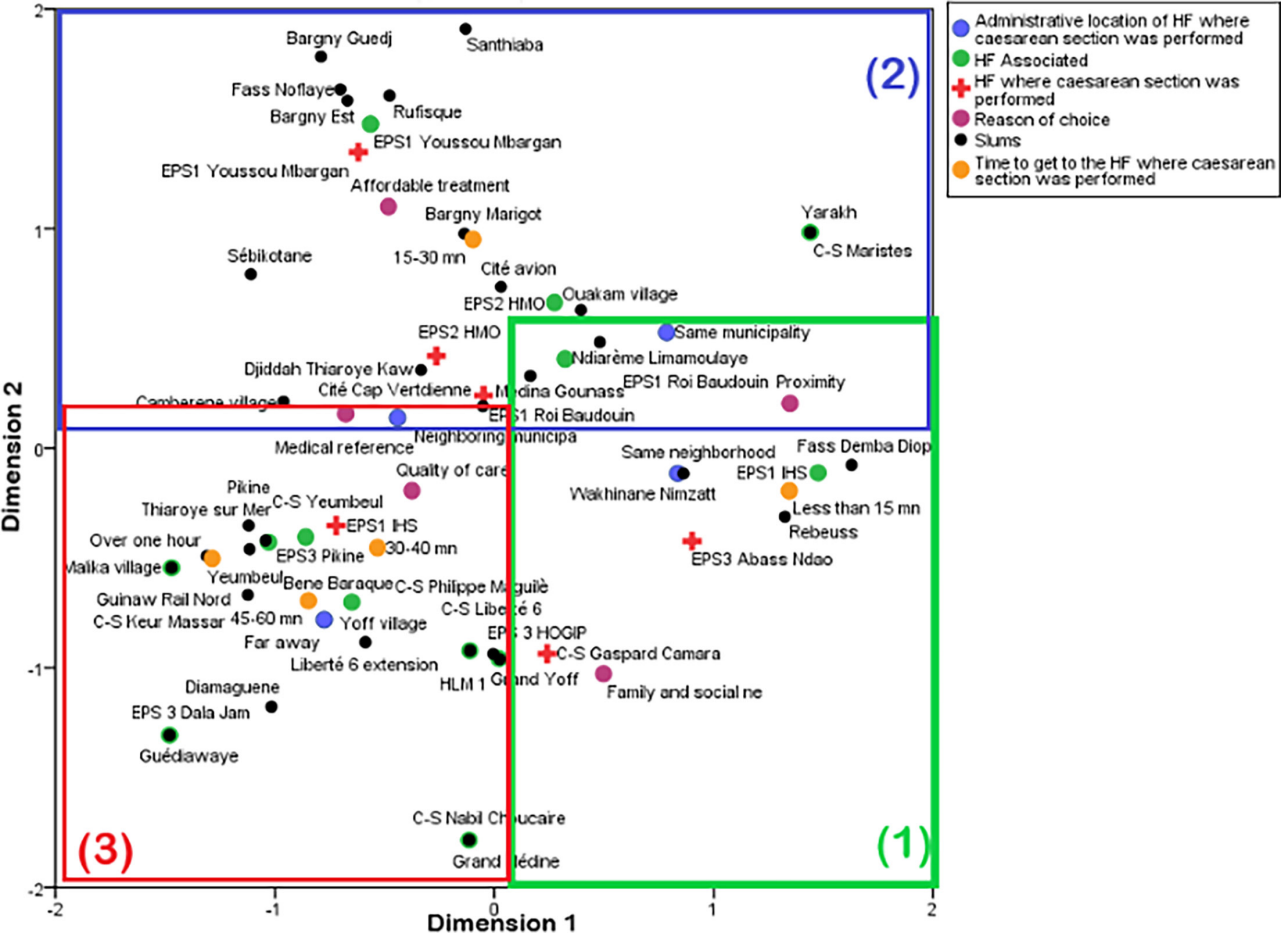
Multiple correspondence factor analysis.

The MCA (figure 4) confirms the bivariate analysis and provides deeper insight into the factors associated with the observed flows. The factorial analysis highlights three major trends:

The first trend focuses on slums located in the health district of Dakar South (eg, Fass and Rebeuss). These areas are characterised by a high density of health facilities performing caesarean sections. The geographical proximity of these health facilities is the primary factor explaining the women’s choice to use them. These women typically spend less than 15 min travel to undergo their caesarean section, often without leaving their municipality, and in some cases, even their neighbourhood.

The second trend involves three health facilities which effectively attract women from nearby slums. Few women living near these facilities bypass them to seek care elsewhere. These facilities also serve as medical referral points for women coming from other municipalities. Women who had a caesarean section at these facilities typically spent between 15 and 30 min travelling.

The third trend involves slums concentrated in the suburbs and other densely populated neighbourhoods of Dakar. This trend is characterised by flows where women bypass nearby health facilities to seek care at CS Gaspard Camara or health facilities in the Dakar South district. Women affected by these flows often spend more than 30 min, and sometimes more than an hour, travelling to reach the facility where they had their caesarean section. Medical referral is the primary factor explaining this trend. These areas are marked by a high population density, and the available health facilities performing caesarean sections often lack sufficient capacity. In emergency cases, these facilities refer parturients to higher level hospitals or those with available beds. Reasons for bypassing may also stem from family or social networks, or from women’s belief that health facilities in Dakar provide superior quality care for childbirth.

## Discussion

This paper examines the geographical accessibility of caesarean care in health facilities near slums in the Dakar region that offer caesarean sections. The first key finding reveals that the majority of slums are located within 6.3 min of motorised travel time (3.8 km along the road network) of a health facility providing caesarean sections. This indicates good geographical accessibility, as women can access these services within 30 min. This result aligns with findings from a study, which also demonstrated the relatively high level of access to caesarean section services in the slums of the Senegalese capital.^23 30^

This accessibility can be attributed to efforts by the Senegalese government since 2005 through the free caesarean section policy, which aims to overcome the physical and financial barriers that have often made this essential obstetric service inaccessible to disadvantaged populations. Additionally, this policy is supported by the National Health and Social Development Plan, which sets each decade the priority areas of health intervention for the State of Senegal.

Despite the good geographical accessibility, the majority of women bypass health facilities offering caesarean sections that are closer to them in favour of those further away. Several factors contribute to this bypass, the primary reason being the medical referral. Contrary to what one might assume, there is no formal referral system for caesarean sections in the Dakar region. All health centres with a functional operating room and expertise in obstetrics and gynaecology are capable of performing this procedure. However, referrals only occur when the facility where the patient initially goes does not have available space or when its operating room is not functional. Therefore, it is usually the patients themselves who decide where to go for their caesarean section, sometimes with advice from nursing staff, based on criteria such as the reputation of the facility, the perceived quality of care, or geographical proximity.

**Figure 5:**
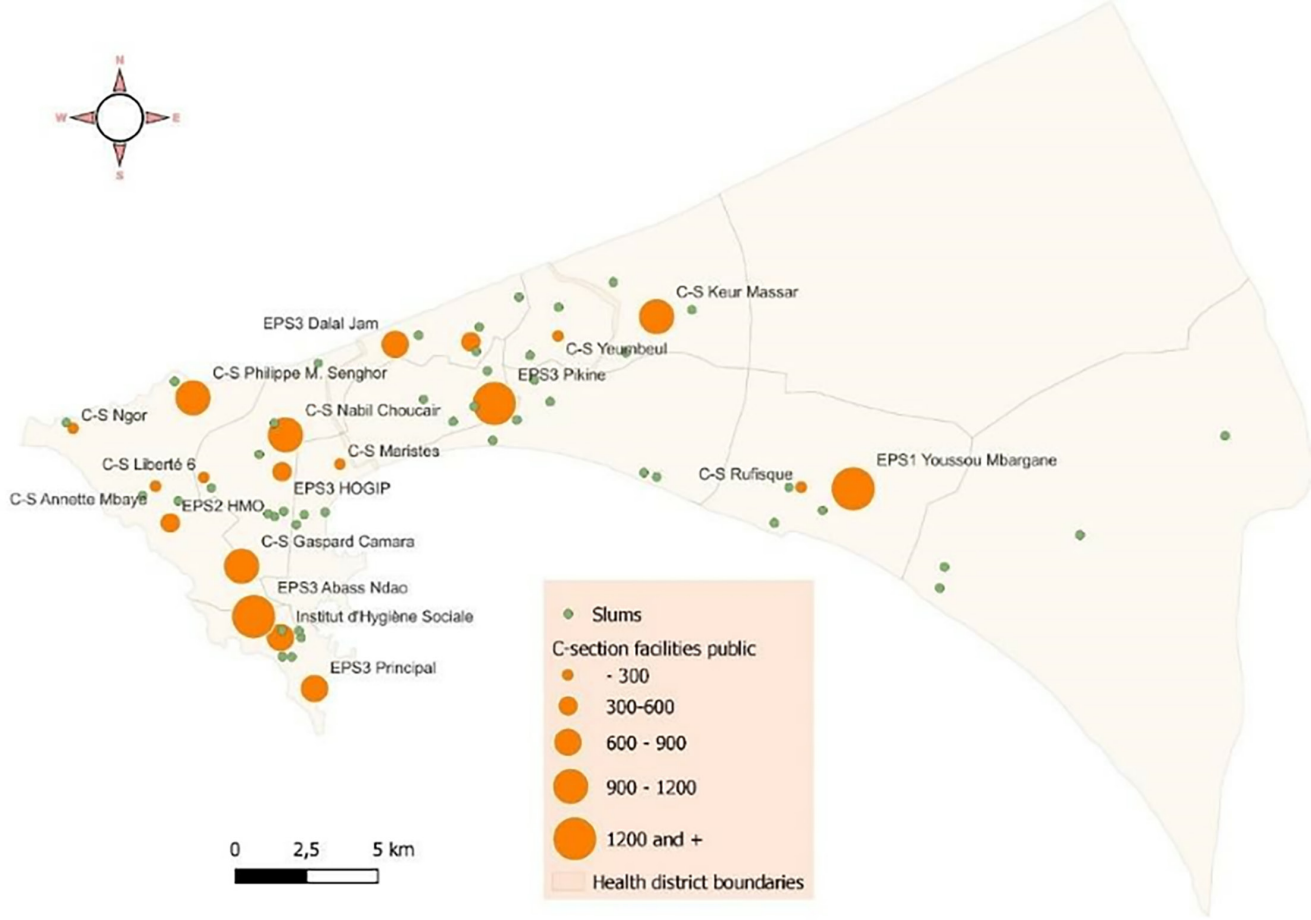
Number of caesarean sections performed in public health facilities in Dakar in 2022 (data source: Dakar medical region, 2022 data, cartographic processing, authors).

For example, the medical referrals observed in the slums served by EPS 3 Pikine (district of Pikine) can be attributed to the high demand for services at this facility, regardless of the women’s place of residence, while the number of available beds is limited. According to data from the Dakar medical region (figure 5), EPS Pikine recorded the highest number of vaginal deliveries (3334) and caesarean sections (2144) in Dakar in 2022. This totals 5478 deliveries, or 15 per day (including weekends), with six caesarean sections and nine vaginal deliveries. However, EPS 3 Pikine had only 35 beds for parturients in the obstetrics department and six gynaecologists at the time of our survey. Since women who undergo a caesarean section must stay in the facility for at least 3 days, and those who deliver vaginally can stay for 2 days, EPS Pikine is often forced to shorten the length of stay for new mothers. This leads to the referral of patients to other health facilities with available beds. This situation highlights the high demand for EPS 3 Pikine, which can lead health posts to refer caesarean section cases to other facilities and, conversely, women bypassing EPS Pikine in favour of less crowded facilities offering higher quality care. A similar pattern is observed in health centres located in densely populated areas, which each performed between 900 and 1200 caesarean sections in 2022, more than several hospitals.

The fact that women bypass nearby health facilities in favour of more distant ones can be explained by the concept of spatial autocorrelation, as outlined by Filiponi and Manghera,^31^ who argue that two nearby locations are more likely to share similar characteristics than two distant ones. Furthermore, it is evident that the flow of women tends to head towards health facilities in the Dakar district, rather than the other way around. These facilities, therefore, attract women living in the slums, highlighting the central role of health services in more urbanised areas.

The phenomenon of women bypassing the nearest health facilities for childbirth is not limited to Dakar and its slums; similar patterns have been observed in other parts of Africa and Asia. In Ghana, for instance, women often bypass nearby health facilities that provide poor quality of care, choosing instead to travel to hospitals much further away that offer better services. Their decision is often based on the availability of qualified personnel and essential medicines, and they are willing to spend more than those who choose local facilities.^32^ A study by Dotse-Gborgborts *et al*^18^ in the Eastern region of Ghana found that 56% of women were willing to spend more than 2 hours on the road to give birth in higher level hospitals, rather than using the nearest primary health facility.

Similarly, in Tanzania, numerous studies have highlighted the tendency of women, particularly from rural areas, to bypass primary health facilities in favour of government hospitals or private religious clinics.^10 14 33^ The likelihood of bypassing increases for primiparous women, those with complications^13^ or those who perceive poor quality of care at the nearest facility. However, this risk decreases if the health facility has recently been renovated or has functional EmONC (Emergency Obstetric and Newborn Care) services.^14^ In rural Mozambique, women who bypassed health facilities tended to be older or from wealthier social backgrounds.^12^

In Kenya, the tendency of bypassing health facilities is more common among urban women, who avoid nearby facilities due to poor quality of care and instead opt for higher level hospitals further away.^23^ In contrast, women from rural or remote areas generally choose institutional delivery only if the nearest health facility is located within 2 km.^34^

Beyond quality of care and geographical proximity, financial factors can also influence the decision to bypass a health facility. A study by Gauthier and Wane^35^ in Chad examined the individual behaviour of women who bypass health facilities and the factors influencing their choice. The findings indicated that poorer women bypass high-quality health facilities in favour of those they can afford, while wealthier women bypass lower quality facilities in search of better care.

The phenomenon of bypassing nearby health facilities for childbirth is widely documented in Asia and Africa. A study by Tappis *et al*^36^ in Afghanistan shows that although many women still give birth at home due to distance and transportation challenges, nearly 60% of women who had skilled assistance during their last birth bypassed the nearest primary care facility. They either delivered in a facility of the same level (10%) or travelled to more distant public or private primary hospitals (60%). In rural Nepal, more than half of the women surveyed by Shah^9^ bypassed the nearest maternity wards to give birth in higher level hospitals. In India, 37.7% of women in a study by Salazar *et al*^37^ bypassed the nearest health facility for delivery.

As in Africa, women in Asia who bypass their local health facilities are often older, first-time mothers or belong to wealthier social categories. Common reasons for bypassing nearby facilities include poor quality of care at the nearest maternity hospitals, such as the absence of an operating room, lack of blood transfusions, insufficient medicines, unqualified personnel and the dilapidated condition of the facilities.^38^ In India, the quality of healthcare staff also affects significantly the likelihood of bypassing, with women more likely to bypass facilities that lack qualified personnel.^11^ A similar trend was observed in Japan by Aoki *et al*,^39^ who demonstrated that patients’ experiences with their primary care physicians correlate with the likelihood of bypassing them in favour of higher level healthcare facilities.

Bypassing does not only affect women and childbirth but also other social categories and types of care. For example, a study in northern Uganda^40^ and China by Li *et al*^41^ highlights the bypass phenomenon in general healthcare, especially among older adults. In China, 40% of people aged 45 and above bypass primary care facilities to access higher level healthcare, often due to factors such as long travel times, higher levels of education, poor health, bad experiences at primary care facilities and the perception of better care in higher level health facilities.^42^

These findings raise questions about the geographical accessibility of health infrastructures, especially in emergency cases. Health facilities must be close to populations to prevent avoidable deaths due to delays in receiving quality care. While travel time estimates are commonly used to assess geographic access to various healthcare services, such as palliative care,^43^ emergency obstetric and neonatal care,^9^ mental health services,^44^ mammography centres^45^ and cervical cancer screening,^46^ the bypass phenomenon challenges the theory that proximity to care services ensures access. It also illustrates the difficulty in finding an appropriate methodology to study healthcare accessibility. The multiple iterations of the floating catchment area method underscore these challenges.^45^ ^47^ ^48^ ^46^

While bypassing nearby facilities might seem intuitively risky for maternal and fetal health due to longer travel times, the situation is more nuanced. Most caesarean sections are planned in advance, with women informed beforehand if they require the procedure due to factors like fetal malposition, maternal comorbidities or other complications. Planned caesareans are typically performed at 37 or 38 weeks of gestation, reducing the risk of going into labour before the procedure. In emergency situations where labour has already started, women are generally cared for at the facility where they first present, minimising critical delays in care.^49^

Although travel time generally does not pose a significant risk in these planned scenarios, the phenomenon of bypassing facilities highlights perceived disparities in the quality of care for vulnerable populations, such as slum residents. Addressing this issue requires improving standards of care in peripheral health centres and ensuring consistent quality of care and patient reception across all facilities. Equipping these facilities with well-trained staff, supported by ongoing professional development, can help build confidence in the care they provide. Additionally, integrating patient feedback mechanisms would enable health administrators to identify areas for improvement in underutilised facilities, thereby fostering a patient-centred approach to enhancing healthcare.^18^

Travel times can be self-reported or modelled. Self-reported travel can be qualitative or quantitative. For instance, the DHS reports perceived distance as a challenge to healthcare accessibility. Also, women visiting a health facility could be asked about the time or distance they travelled. Comparatively, modelled travel times rely on using geographic topological data or censor-based data to estimate travel time and distance. Comparatively, self-reported or perceived travel time estimates are generally longer compared with modelled ones.^50^ This may explain why self-reported travel times (figure 4) are slightly higher than modelled travel times (figure 1) in this study. Furthermore, women in urban areas might find distance to care more challenging than rural women, although rural women travel longer to seek care.^51^ Therefore, this context should be considered when comparing our bypassing categories based on modelled travel times with self-reported travel times.

### Limitation

Our geographic accessibility estimation has some limitations. For medical referrals, the mapping analysis did not consider referral times/distance between home and the first health facility to which the woman went, and then referral from this facility to the health facility where the caesarean section was performed. This may underestimate the challenges of accessibility for referred women. Furthermore, although we estimated travel times, our model does not take into account the real-time traffic, which could be challenging in urban settings such as Dakar.^52^ In terms of the design of the study, exclusion of women living outside slums could be a limitation. In fact, the inclusion of women living in middle-income and affluent neighbourhoods would have added more value to the analyses, as bypassing health facilities offering caesarean sections may not be specific to slums, but may be a widespread public health problem in Senegal. The analyses did not consider women’s sociodemographic and socioeconomic characteristics, as many studies have done, to show the profiles of women who tend to bypass CEmONC facilities. Although slums are a homogeneous environment and women may share the same sociodemographic characteristics, a study that takes this factor into account, as well as other types of urban neighbourhood, could be relevant. Finally, the article focuses only on caesarean sections, which limits the generalisability of the results to other forms of obstetric or medical care.

### Implication

The broader implications of bypassing health facilities on maternal-foetal well-being deserve further discussion. Bypassing can result in significant delays in accessing timely care, especially for women experiencing obstetric emergencies. Longer travel times are associated with increased risks of adverse outcomes, including maternal mortality, foetal distress, stillbirth and neonatal complications. These risks are compounded in slums with limited transportation infrastructure, where delays in reaching higher level facilities are often inevitable.

In terms of recommendations, we can propose the following strategies for the government to address this issue:

- Strengthening the referral system: the government could improve the effectiveness and efficiency of the referral system by ensuring that lower level facilities are equipped and by enhancing communication and transportation between facilities.
- Improving quality of care in primary-level facilities: addressing perceived or actual gaps in the quality of care at local health facilities could reduce the need for women to bypass these facilities. Investments in staff training, equipment and the availability of essential medications could help build trust in primary-level care.
- Expanding emergency transport services: establishing or strengthening emergency medical transport systems, such as ambulances or community-based transport networks, could reduce delays for women needing to reach higher level facilities.
- Increasing awareness and education: community-level interventions to educate women and families about the importance of seeking care at the nearest facility in emergencies could help reduce unnecessary bypassing.

## Conclusion

In Dakar, despite good access to health facilities performing caesarean sections, the phenomenon of women bypassing nearby facilities for reasons beyond their control deserves special attention. Bypassing these health facilities leads to longer travel times, higher costs for women and their families and inefficiencies in the healthcare system. The primary factors driving this bypass, medical referrals and seeking better quality care, reflect significant dysfunctions in the healthcare system. These issues can be attributed to poor quality of care, limited capacity of local facilities and lack of qualified healthcare personnel in facilities closer to the slums.

This study makes several important contributions. First, it focuses solely on the bypassing of health facilities for caesarean sections, whereas previous studies focused mainly on general delivery without specifying the type of delivery. Second, it highlights the particular difficulties faced by women in disadvantaged urban neighbourhoods, as opposed to the more commonly studied rural populations. Finally, the study identifies the underlying reasons for bypassing through both women’s responses and multivariate analyses. This makes it one of the few studies to show that medical referrals are a significant factor behind bypass, suggesting that the issue is not solely under women’s control.

To address the issue of bypassing in urban slums, it is crucial to improve the quality of care, increase the capacity of nearby health facilities and ensure the presence of qualified healthcare personnel to meet the needs of women in these underserved areas. Additionally, improving the medical referral system and investing in the infrastructure of local health centres can reduce the need for women to travel long distances, ensuring timely access to essential obstetric services.

## Data Availability

Data are available upon reasonable request.
