# Supplementary material for: Beyond physical accessibility, bypassing health facilities offering caesarean section: insights from women in Dakar’s slums
Source: BMJ Open. 2025 Mar 22;15(3):e088606. doi: 10.1136/bmjopen-2024-088606 (PMC11931907; doi:10.1136/bmjopen-2024-088606)
Supplement: Supplementary file 1 [file bmjopen-2024-088606.reviewer_comments.pdf]

PEER REVIEW HISTORY

BMJ Open publishes all reviews undertaken for accepted manuscripts. Reviewers are asked to complete a checklist review form and are provided with free text boxes to elaborate on their assessment. These free text comments are reproduced below.

ARTICLE DETAILS

Title (Provisional)

Beyond physical accessibility, bypassing health facilities offering cesarean section: Insights from Women in Dakar's Slums

Authors

Sylla, El Hadji Malick; Fall, Ndeye Awa; Dotse-Gborgbortsi, Winfred; SANDIE, Arsène Brunelle; Gueye, Barrel Sow; Senghor, Diarra Bousso; Cissé, Birane; Bocoum, Fadima Yaya; Sy, Ibrahima Ousmane; Faye, Cheikh

---

VERSION 1 - REVIEW

**Reviewer**	**1**
**Name**	**Ravit, Marion**
**Affiliation**	**Universite Paris Descartes**
**Date**	**04-Jul-2024**
**COI**	**I declare no competing interests**

This paper presents a geographical analysis of women in the slums of Dakar who need a caesarean section and how they can access care. It is interesting to note that most of these women access care easily when they need a caesarean. However, more than two-thirds of them bypass the nearest health facility to have an intervention in a health facility further away.

This is a very notable and interesting way of studying primary healthcare and caesarean section in sub-Saharan Africa, a subject that has been widely studied. It also looks at access to care, providing a very useful and interesting geographical analysis.

However, the fact that caesarean is specifically studied here is not justified. We need more information on the rate of caesarean in Senegal, why this is a particular issue. We also need more context, particularly in relation to free healthcare policy, how it might impact on access to healthcare for women.

In addition, the methodology needs to be explained more clearly, particularly the sample selection procedure and the different types of data used.

Introduction

Page 2: Please include more information on caesarean section (CS) in Senegal, (statistics, procedure, policies …) especially in context of free health care. The literature on CS in Senegal is very vast.

Methods

Page 3 line 9-10: what is a low CS rate? Why excluded these districts for this reason although you can observe which women are concern in this specific health district

Page 3 lines 29 30: What data do you use to support your statement that 80% of CS is carried out in these establishments?

Page 3 lines 32-33: On what basis do you say that women from the slums do not use private facilities? Aren't they covered by the free policy?

Page 3 line 40: How are these women selected? Randomly? Please give us more information on the total number of women who gave birth in all facilities during the period.

Page 4 line 21: Who are these 747 women? What are "flows"? Could you give us a definition?

Results

page7 line 49: Observed flow concern how many women ? 747 or 108?

Page 8 line 40: 65.5% (it is from 108 women?)

Page 9 figure 5: please add the totalnumber of women

Page 9 line 29: please refer to figure 5

Page 10 figure 6: please specify how many women are affected in total (108 or 747?) and by hospital

Page 11: Do you have any information on whether women who have bypassed the nearest health center have previously attended another facilities? For example, a woman may have gone to a health facility that does not perform caesarean sections and then been referred to another facility for a caesarean section.

Discussion

Page 13 lines 20-50: Do you have any references that can corroborate your hypothesis about the reference system?

Page 13 line 33: figure 8 is mentioned but not available in the pdf.

Page 15 lines 37-52: I think that in your study, the main concern is not geographical accessibility but the quality of care in health establishments, the efficiency of the health system and the way the referral system works. The situation may be different in rural areas, and you should explain this.

Other comments:

You should add a limitation paragraph (no national representativeness, no specific information on women's backgrounds, small sample…).

**Reviewer**	**2**
**Name**	**Dashraath, Pradip**
**Affiliation**	**National University Hospital**
**Date**	**18-Aug-2024**
**COI**	**Nil**

Thank you for inviting me to review this cross-sectional retrospective study on the physical accessibility for women in Dakar to health establishments performing cesarean section. This is a highly detailed geographical study which provides some insights into the health seeking behaviour of pregnant individuals living in slums.

My main comment concerns a point of clarification: could the authors please explain if a formal referral system to hospitals exists? This information is important to place Figures 2 and 3 into context because the expected movement of people is assumed to be only based on physical proximity. However, from a reader's perspective, it appears that a medical referral system is in place (which will result in patients bypassing the nearest healthcare facility), given that nearly 65% of women from the non-bypassing group mention medical referral as their reasons (presumably because it is a high-risk pregnancy or the woman requires a higher level of medical care). If this is not the case, please explain this clearly in the article.

Additionally, about 30% of women choose C-S Gaspard Camara and nearly 50% choose EPS3 Abass Ndao (rather than the nearer EPS3 HOGIP) because of 'proximity'. However, based on Figure 3, it seems there would not be women visiting these centres, given that the distance is more than 15 minutes travelling time as per the author's multiple correspondences factorial analysis in Figure 7. Why then have they listed proximity as the reason for choosing these centres? Please clarify.

Please also define terms such as "far away," "same municipality," and "same neighbourhood" You may consider elaborating in the methodology section (or with a diagram) which slums comprise a single neighborhood etc. Further, the use of standardized units of measure, eg kilometers, should be used in place of 'far away' for Table 1.

Finally, please include in the discussion more detail regarding the broader implications on maternal-fetal wellbeing of bypassing health facilities, eg the occurrence of adverse obstetric outcomes due to travelling time. How might you propose the government addresses this?

**Reviewer**	**3**
**Name**	**Macharia, Peter**
**Affiliation**	**Institute of Tropical Medicine, Department of Public Health**
**Date**	**23-Sep-2024**
**COI**	**None**

Data

1. The data from private clinics were excluded due to low use by women living in slums. What was this % that warranted the exclusion of private facilities in the analysis?

2. It's not clear where the number 757 women comes from in relation to the number of surveyed women (260).

3. Also related to the preceding point you cannot exclude facilities so that a map is not overloaded (Figure 3 and 4 justification) as indicated. You should plot the maps on panels and zoomed-in sections to show all necessary information.

4. Were the data from Dakar regions in 2022 on vaginal and cesarean deliveries disaggregated by month, if not how were they matched to the six (6) months surveys (July to December 2022)

Spatial access

5. My major concern is around cartographic analysis, it shows straight-line distances were used. These straight-line distances have major assumptions as summarized in Ouma et al 2021. " That travel occurs in a straight line and ignores the influence of transport services on accessibility and barriers of travel such as land use, road network and elevation". Consider using alternative approaches

6. Further travel time is a preferred measure relative to distance because time accounts for mode of transport and barriers while distance does not. For example, the said 2km in the manuscript could be 24 minutes walking but 2 minutes driving. Without accounting for a mode of transport, several biases are inherent

7. The cartographic analysis also does not seem to account for referral times/distance that's from home to facility A and then referral from facility A to major facility

8. The flows in Figure 3 are still based on straight lines, which does not apply in the real world due to transport barriers that should be accounted for in this analysis

9. Some travel time estimates are indicated in section 3.3.1. It's not clear whether these were modelled or were self-reported and how they related to the modelled straight-line distance

10. Also the concept of perceived travel time and modelled travel time needs to be discussed as it is missing in the paper

11. Will the publishing of actual facility names be an ethical issue when located together with the reason of bypassing and not bypassing by women?

12. When women are referred to higher facilities for CS, is that bypassing? they did not do this out of their own volition but due to medical reasons initiated by a facility. A sensitivity analysis will be useful in looking at the data having excluded those who were referred due to medical reasons

13. The limitations of the analyses have not been discussed.

14. Slums are shown as dots (should be an area, not a dot) on the maps, is it possible then that some women and facilities are not located in the slums? Does this mean the analysis is a mixture of women from both slums and non-slums?

Ouma, P., Macharia, P.M., Okiro, E., Alegana, V. (2021). Methods of Measuring Spatial Accessibility to Health Care in Uganda. In: Makanga, P.T. (eds) Practicing Health Geography. Global Perspectives on Health Geography. Springer, Cham. https://doi.org/10.1007/978-3-030-63471-1_6

VERSION 1 - AUTHOR RESPONSE

**Reviewers**	**comments**	**answer**
Reviewer 1	Introduction<br><br>Page 2: Please include more information on caesarean section (CS) in Senegal, (statistics, procedure, policies …) especially in context of free health care. The literature on CS in Senegal is very vast.	More literature on c-section in Senegal was added.
	Methods<br><br>Page 3 line 9-10: what is a low CS rate? Why excluded these districts for this reason although you can observe which women are concern in this specific health district	Yes, we could look at these women in these health districts, but they are very remote districts and we had limited means to enlarge our sample.
	Page 3 lines 29 30: What data do you use to support your statement that 80% of CS is carried out in these establishments?	We have reviewed the sentence.
	Page 3 lines 32-33: On what basis do you say that women from the slums do not use private facilities? Aren't they covered by the free policy?	We have reviewed the sentence.

	Page 3 line 40: How are these women selected? Randomly? Please give us more information on the total number of women who gave birth in all facilities during the period.	We provided additional information on that
	Page 4 line 21: Who are these 747 women? What are "flows"? Could you give us a definition?	Additional information is provided in the text to clarify these points.
	Results<br>page7 line 49: Observed flow concern how many women ? 747 or 108?	It concerns 763 women, we have made the change in the text
	Page 8 line 40: 65.5% (it is from 108 women?)	Yes
	Page 9 figure 5: please add the total number of women	Done
	Page 9 line 29: please refer to figure 5	Done
	Page 10 figure 6: please specify how many women are affected in total (108 or 747?) and by hospital	Done<br>At page 10
	Page 11: Do you have any information on whether women who have bypassed the nearest health center have previously attended another facilities? For example, a woman may have gone to a health facility that does not perform caesarean sections and then been referred to another facility for a caesarean section.	Thanks for your comment, unfortunately we do not have this information . Our focus was on the health facility where women gave birth by C-section.
	Discussion<br>Page 13 lines 20-50: Do you have any references that can corroborate your hypothesis about the reference system?	It was added into the text
	Page 13 line 33: figure 8 is mentioned but not available in the pdf.	All the figure are now available
	Page 15 lines 37-52: I think that in your study, the main concern is not geographical accessibility but the quality of care in health establishments, the efficiency of the health system and the way the referral system works. The situation may be different in rural areas, and you should explain this.	Done<br>We revised the explanation in the followed sentence: “.......

	Other comments:<br>You should add a limitation paragraph (no national representativeness, no specific information on women's backgrounds, small sample…).	A limitation paragraph has added in the discussion section
Reviewer 2	could the authors please explain if a formal referral system to hospitals exists? This information is important to place Figures 2 and 3 into context because the expected movement of people is assumed to be only based on physical proximity. However, from a reader's perspective, it appears that a medical referral system is in place (which will result in patients bypassing the nearest healthcare facility), given that nearly 65% of women from the non-bypassing group mention medical referral as their reasons (presumably because it is a high-risk pregnancy or the woman requires a higher level of medical care). If this is not the case, please explain this clearly in the article.	Done
	Additionally, about 30% of women choose C-S Gaspard Camara and nearly 50% choose EPS3 Abass Ndao (rather than the nearer EPS3 HOGIP) because of 'proximity'. However, based on Figure 3, it seems there would not be women visiting these centres, given that the distance is more than 15 minutes travelling time as per the author's multiple correspondences factorial analysis in Figure 7. Why then have they listed proximity as the reason for choosing these centres? Please clarify.	New figures have been introduced to take account of these issues
	Please also define terms such as "far away," "same municipality," and "same neighbourhood" You may consider elaborating in the methodology section (or with a diagram) which slums comprise a single neighborhood etc. Further, the use of standardized units of measure, eg kilometers, should be used in place of 'far away' for Table 1.	We've removed this part, which was confusing. We used the modelled travel time
	Finally, please include in the discussion more detail regarding the broader implications on maternal-fetal wellbeing of bypassing health facilities, eg the occurrence of adverse obstetric outcomes due to travelling time. How might you propose the government addresses this?	Done

Reviewer 3	The data from private clinics were excluded due to low use by women living in slums. What was this % that warranted the exclusion of private facilities in the analysis?	We have reviewed the methodology. Only data from public health institutions are used.
	It's not clear where the number 757 women comes from in relation to the number of surveyed women (260).	We have provided clarifications on the methodology
	Also related to the preceding point you cannot exclude facilities so that a map is not overloaded (Figure 3 and 4 justification) as indicated. You should plot the maps on panels and zoomed-in sections to show all necessary information.	Thanks for the suggestion. We have now put figures 3 and 4 into one figure of two panels as you suggested.
	Spatial access<br>5. My major concern is around cartographic analysis, it shows straight-line distances were used. These straight-line distances have major assumptions as summarized in Ouma et al 2021. " That travel occurs in a straight line and ignores the influence of transport services on accessibility and barriers of travel such as land use, road network and elevation". Consider using alternative approaches	We have revised our approach to estimating geographic accessibility. We agree that straight line distances are too simplistic for the study area. Therefore, we have improved our estimation by using a network-based motorised travel time model.
	Further travel time is a preferred measure relative to distance because time accounts for mode of transport and barriers while distance does not. For example, the said 2km in the manuscript could be 24 minutes walking but 2 minutes driving. Without accounting for a mode of transport, several biases are inherent	We have now used travel time estimates to measure proximity.
	The cartographic analysis also does not seem to account for referral times/distance that's from home to facility A and then referral from facility A to major facility	Referral times/distance did not take into account because there is no sufficient information about the facilities they visited prior.
	The flows in Figure 3 are still based on straight lines, which does not apply in the real world due to transport barriers that should be accounted for in this analysis	We have improved our flow maps and travel time estimation in figure 2.
	9. Some travel time estimates are indicated in section 3.3.1. It's not clear whether these were modelled or were self-reported and how they related to the modelled straight-line distance	We revised it. Now, It is only the travel time in the ACM that is self-reported and this allows comparison with the modelled travel time.
	10. Also the concept of perceived travel time and modelled travel time needs to be discussed as it is missing in the paper	Thanks for the suggestion. We have added a paragraph to the discussion to highlight the importance of considering self-

		reported and modelled travel time estimates. We have added relevant references.
	11. Will the publishing of actual facility names be an ethical issue when located together with the reason of bypassing and not bypassing by women?	It's relevant, but it's not a problem in Senegal. One of the authors of the article is the current Minister of Health
	12. When women are referred to higher facilities for CS, is that bypassing? they did not do this out of their own volition but due to medical reasons initiated by a facility. A sensitivity analysis will be useful in looking at the data having excluded those who were referred due to medical reasons	We have now included this as a limitation to our geographic accessibility method.
	13. The limitations of the analyses have not been discussed.	We have now added a paragraph to discuss the limitations.
	14. Slums are shown as dots (should be an area, not a dot) on the maps, is it possible then that some women and facilities are not located in the slums? Does this mean the analysis is a mixture of women from both slums and non-slums?	Thanks for this comment. Considering the scale at which we are analysing data, we chose to use central points that represent the slum communities. Also, the origin-destination tools we used to estimate the travel times only accepts point inputs.
